# Centroid Migration and Distribution of Dominant Species in Different Grassland Types Revealing Climate Change Responses on the Qinghai–Tibet Plateau

**DOI:** 10.3390/plants15131972

**Published:** 2026-06-26

**Authors:** Wen-Wen Guo, Wen-Long Li, Wen-Ting Wang, Wen-Ying Wang, Hua-Kun Zhou, Jing Xu, Xing-Yuan Liu, Si-Qing Li

**Affiliations:** 1 State Key Laboratory of Herbage Improvement and Grassland Agro-Ecosystems, College of Pastoral Agriculture Science and Technology, Lanzhou University, Lanzhou 730030, China; xiaowzye@163.com (W.-W.G.); 220220902460@lzu.edu.cn (X.-Y.L.); lisq2024@lzu.edu.cn (S.-Q.L.); 2 School of Mathematics and Computer Science, Northwest Minzu University, Lanzhou 730030, China; iamwwt1983@163.com; 3 Department of Life Sciences, Qinghai Normal University, Xining 810008, China; wangwy0106@163.com; 4 Qinghai Provincial Key Laboratory of Restoration Ecology for Cold Region, Northwest Institute of Plateau Biology, Chinese Academy of Sciences, Xining 810008, China; hkzhou@nwipb.cas.cn; 5 School of Agriculture and Forestry Economic and Management, Lanzhou University of Finance and Economics, Lanzhou 730020, China; xujing@lzufe.edu.cn

**Keywords:** Qinghai–Tibet Plateau, suitable habitat, ensemble model, dominant species, climate change

## Abstract

The Qinghai–Tibet Plateau (QTP) is highly sensitive to global climate change, and the stability of its grassland ecosystems is critical for regional ecological security and livestock development. Therefore, investigating future spatial distribution changes of dominant species on the QTP is of great importance for grassland management. In this study, an ensemble model was used to simulate and analyze the potential distribution and centroid migration directions of dominant species in alpine meadow, alpine grassland, desert grassland, and temperate grassland under current and future climate scenarios (SSP2-4.5 and SSP5-8.5). The results show that the ensemble model achieved excellent predictive accuracy for all species (AUC > 0.9, TSS > 0.7, Kappa > 0.6). Elevation is the key factor governing species distribution, while climate drivers differ significantly among species. The distribution of dominant species in alpine meadow and alpine grassland is primarily co-driven by the mean monthly temperature range (MTR), isothermality (ISO), and annual precipitation (AP); desert grassland dominants are mainly influenced by AP and the mean temperature of the driest quarter (MTDQ); and temperate grassland dominants are driven by the precipitation of the coldest quarter (PCQ) and AP. The suitable habitats of dominant species in the future will generally expand towards high-altitude, high-latitude regions in the north and northwest, with centroid migration directions varying markedly among species. Specifically, the centroids of desert grassland dominants and *S. bungeana* in temperate grassland will migrate northwest under SSP2-4.5 and SSP5-8.5, while *N. splendens* and *S. krylovii* in temperate grassland will migrate southwest. For alpine meadow and alpine grassland dominants, the centroids will generally move northwest under SSP2-4.5 but diverge under SSP5-8.5—*E. nutans* and *S. purpurea* in alpine grassland will continue to migrate northwest, whereas alpine meadow dominants and *P. annua* in alpine grassland will migrate east or northeast. This study provides a theoretical basis for grassland conservation, biodiversity conservation, and livestock production in response to climate change on the QTP.

## 1. Introduction

The Qinghai–Tibet Plateau (QTP) is the largest plateau in the world (covering an area of approximately 2.5 million km^2^), with grasslands accounting for more than 60% of its total area [1,2]. Due to its distinctive geography and climate, the QTP is regarded as one of the most sensitive regions to global climate change [3]. The QTP has already experienced an earlier and faster warming process, which is expected to continue at an accelerated pace in the future [4,5], with the regional climate exhibiting a marked trend towards warmer and wetter conditions [6,7]. Grasslands on the QTP not only serve as the foundation for the sustainable development of plateau animal husbandry but also serve as key carriers for maintaining alpine biodiversity and ecosystem service functions [8,9]. However, with the increasing frequency of global extreme heatwaves and drought events, changes in regional hydrothermal patterns are driving significant shifts in vegetation distribution, posing a serious threat to the stability of terrestrial ecosystems [10,11]. Against this background, investigating the responses and habitat suitability dynamics of different grassland types on the QTP to future climate change is not only a major scientific question in global climate change research but also a practical issue that urgently needs to be clarified for regional pastoral planning and ecological conservation decision-making.

As the core carriers of plant community structure and function, the distribution patterns and dynamics of dominant species are key indicators for revealing trends in ecosystem evolution [12]. Dominant species not only determine the structural characteristics of plant communities but also profoundly affect ecosystem service functions [13]. Focusing on dominant species can simplify complex ecological research and provide a critical entry point for studying grassland ecosystems under climate change [12,14]. Existing studies indicate that the spatial distribution areas of dominant species in alpine meadow and alpine grassland on the QTP account for approximately 76% and 92% of the total alpine grassland area, respectively [15]. Bai and Hou (2021) examined the impact of climate change on *Leymus chinensis*, a dominant grassland plant species in northern China [14]. Hou et al. (2023) reported that dominant species play a leading role in shaping community stability in the northern Tibetan grasslands [16]. Furthermore, climate warming can further enhance the competitive advantage of dominant species by reshaping the pattern of interspecific competition [17,18]. For example, Ma and Sun (2018) found that the distribution range of the alpine grassland dominant species *Stipa purpurea* on the QTP will continue to expand with increasing temperature [19]. Ma et al. (2023) pointed out that the cold- and drought-tolerant biological traits of dominant species are the core mechanisms underlying the response of alpine grassland to climate change [20]. Therefore, studying changes in the distribution patterns of grassland dominant species in the context of climate change can provide a theoretical basis for predicting future trends in grassland vegetation succession.

In recent years, ecological niche modeling (ENM) (e.g., Maxent, random forests, support vector machines, etc.) has gained increasing attention in predicting the impacts of future climate change on species distribution [21] and species invasions [22]. As the predictions of ENMs depend on the actual locations of species occurrences and environmental variables, different models for the same species may yield divergent results [23,24,25]. Currently, ensemble methods, such as multi-model inference and model averaging, have been employed to reduce uncertainties among ENMs, as they have been shown to improve model predictions, reduce overfitting in species modeling, and often outperform single models in predicting species distributions [24,25,26,27]. For example, Canturk et al. employed an ensemble modeling approach to project the potential distributions of *Prunus avium* L. [26], *Laurus nobilis* L. [27], and *Picea orientalis* [28] under future climate change scenarios. Yang et al. (2024) predicted the impacts of future climate change on the distribution and niche of the key afforestation species *Cupressus funebris* through an ensemble model [29]. Amindin et al. (2024) employed an ensemble approach to forecast the future distribution of *Fritillaria imperialis* [30]. However, existing studies have largely focused on the spatial distribution modeling of single or rare species, and systematic research on changes in the distribution of diverse grassland types on the QTP under different future climate scenarios remains scarce.

Based on the above research background, this study proposes the following scientific hypotheses: (1) Elevation is the dominant environmental factor constraining the geographic distribution of species, while the combined effects of temperature and precipitation drive clear niche differentiation among dominant species of different grassland types. (2) Climate warming will promote the expansion of suitable habitats of dominant species towards higher altitudes and higher latitudes in the future. (3) The centroid migration directions of dominant species vary among different grassland types. To test these hypotheses, this study selected 12 dominant species from alpine meadow, alpine grassland, desert grassland, and temperate grassland on the QTP. We then simulated their spatial distribution patterns under current and future climate scenarios through the ensemble modeling of three ENMs, analyzed the dominant factors influencing their distribution, and quantified changes in suitable habitats and centroid shift trajectories. Subsequently, we extrapolated the trends in spatial distribution changes for different grassland types in the future, with the aim of providing a theoretical basis for understanding the response mechanisms of alpine grassland ecosystems to climate change.

## 2. Results

### 2.1. Model Evaluation and Main Driving Factors Affecting the Distribution of Dominant Species

**Figure 1:**
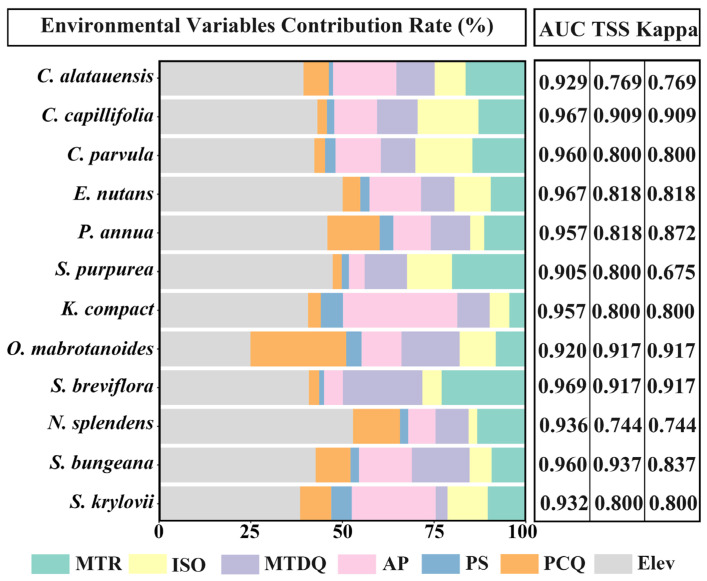
Predicted potential distribution of dominant species across different grassland types under the current climate: environmental variable contribution rate (%) and ensemble model accuracy (AUC, TSS and Kappa). Here, MTR represents mean monthly temperature range, ISO represents isothermality, MTDQ represents mean temperature of the driest quarter, AP represents annual precipitation, PS represents precipitation seasonality, PCQ represents precipitation of the coldest quarter, and Elev represents elevation.

Our results show that the ensemble model exhibited excellent predictive accuracy for the 12 dominant species, with AUC > 0.9, TSS > 0.7, and Kappa > 0.6 for all species (Figure 1). This indicates that the ensemble model, constructed based on the relationship between species occurrence points and environmental factors, has high reliability in simulating species distributions. The contribution rates of environmental variables reveal that elevation is the primary driver affecting the distribution of the 12 species, while the effects of climate variables differ significantly among species of different grassland types, collectively reflecting the adaptive differentiation of species to the hydrothermal gradient on the QTP. Specifically, temperature (Mean monthly temperature range (MTR), Isothermality (ISO), and Mean temperature of the driest quarter (MTDQ)) and Annual precipitation (AP) play a key regulatory role in the distribution of *C. alatauensis*, *C. capillifolia*, *C. parvula* and *E. nutans*; *S. purpurea* and *S. breviflora* exhibit a relatively strong response to variations in temperature (MTR, ISO, and MTDQ); *O. abrotanoides*, *S. bungeana* and *S. krylovii* are primarily driven by AP and precipitation of the coldest quarter (PCQ); AP makes a significant contribution to the distribution of *K. ceratoides*; and the distribution of *P. annua* and *N. splendens* is primarily driven by a combination of temperature (MTR and MTDQ) and PCQ.

### 2.2. Potential Distribution of Dominant Species Under Current and Future Climate Scenarios

**Figure 2:**
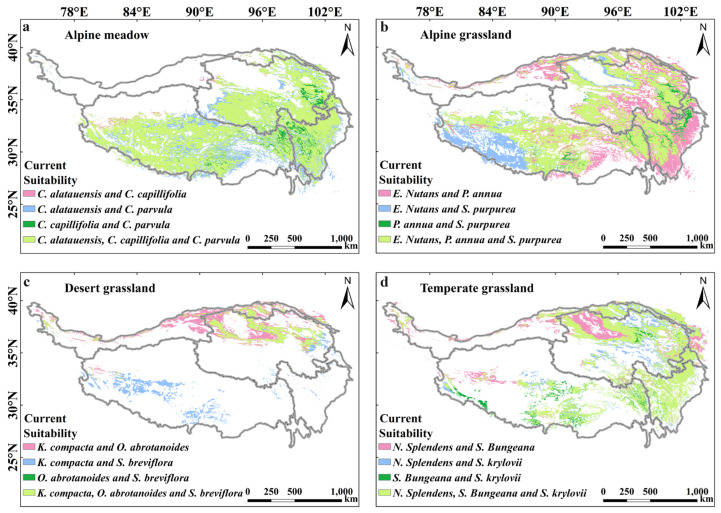
Current suitable habitat distribution patterns of dominant species across different grassland types on the QTP. Among them, subfigure (**a**) represents alpine meadow, (**b**) represents alpine grassland, (**c**) represents desert grassland, and (**d**) represents temperate grassland.

Our results show that the potentially suitable habitats for the dominant species of four grassland types on the QTP exhibit significant spatial heterogeneity under the current climate (Figure 2). The potential distribution overlap of the three dominant species in alpine meadows is the largest, reaching 142.1 × 10^4^ km^2^, mainly concentrated in eastern Tibet, western Sichuan, and southern Qinghai (Figure 2a, Table 1); among them, the area of potential distribution overlap between *C. capillifolia* and *C. parvula* is concentrated in the southeastern QTP, while *C. alatauensis* and *C. parvula* are distributed in a strip-like pattern along the southern QTP. The area of overlap in the potential distributions of the three dominant species in alpine grassland is 105.57 × 10^4^ km^2^, mainly concentrated in the northern Tibetan Plateau and the semi-arid region of southern Qinghai (Figure 2b, Table 1); among them, the overlap in the potential distributions of *E. nutans* and *P. annua* is relatively extensive, whereas *S. purpurea* and *E. nutans* are concentrated in the southwestern QTP. The area of potential distribution overlap for the three dominant species in desert grassland is the smallest (17.32 × 10^4^ km^2^), with a generally fragmented distribution mainly around the northern Tsaidam Basin (Figure 2c, Table 1). The overlap in the potential distributions of *K. compacta* and *O. abrotanoides* is concentrated in the northwestern QTP, while *K. compacta* and *S. breviflora* are mainly distributed in the southwestern QTP. The distribution of temperate grasslands exhibits a pattern of being more abundant in the east and less in the west, with the overlap in the potential ranges of its dominant species (74.44 × 10^4^ km^2^) mainly concentrated in the low-altitude regions of the northeastern QTP (Figure 2d, Table 1). Specifically, the overlap in the potential ranges of *N. splendens* and *S. bungeana* is mainly distributed in the northeastern QTP, while *S. bungeana* and *S. krylovii* are distributed in the southern QTP.

**Figure 3:**
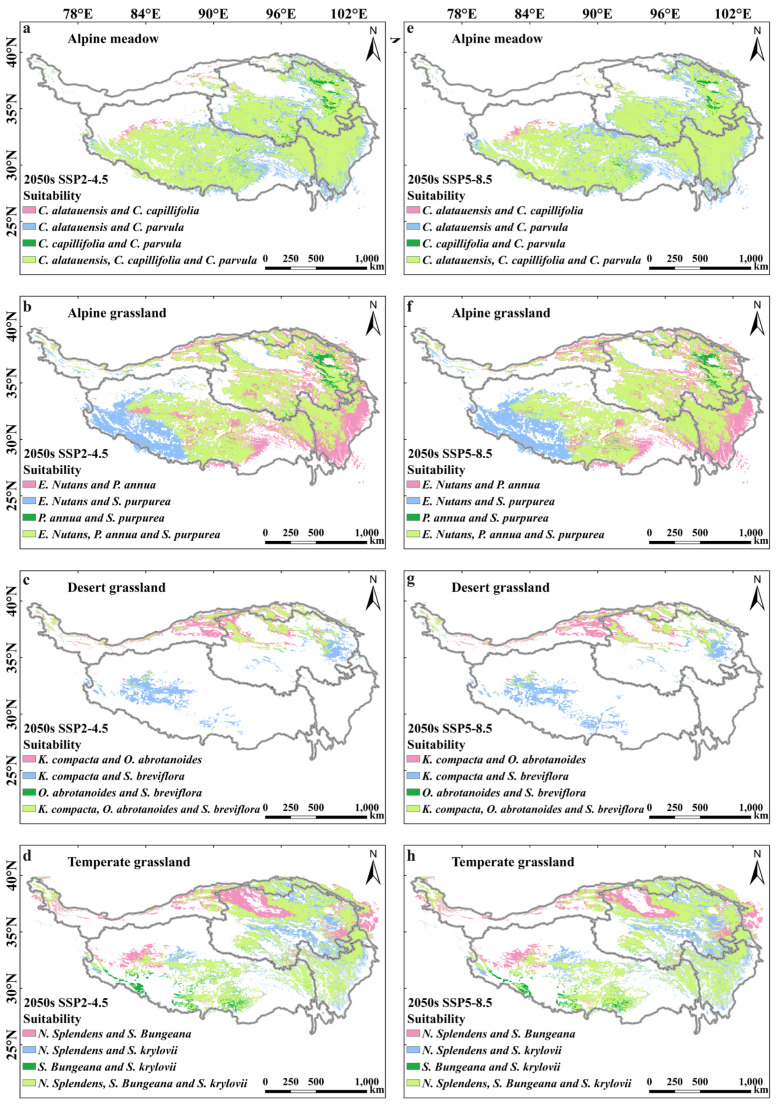
Potential distribution of dominant species across different grassland types on the QTP under future climate change. The columns in the figure correspond to two SSPs (SSP2-4.5 and SSP5-8.5), with subfigures (**a**–**d**) and (**e**–**h**) representing the four grassland types under each scenario: (**a**,**e**) represent alpine meadow, (**b**,**f**) represent alpine grassland, (**c**,**g**) represent desert grassland, and (**d**,**h**) represent temperate grassland.

The suitable habitat patterns of dominant species in four grassland types on the QTP show significant changes under SSP2-4.5 and SSP5-8.5 scenarios (Figure 3). Under the SSP2-4.5 and SSP5-8.5 scenarios, the overlap in the potential distribution of the three dominant species in alpine meadows exhibits an increasing trend from southeast to northwest. Its area will expand to 163.88 × 10^4^ km^2^ (+15.33%) under SSP2-4.5 and contract to 155.73 × 10^4^ km^2^ (+9.59%) under SSP5-8.5, but is still higher than the current level (Figure 3a,e, Table 1). For alpine grassland, the overlap in the potential ranges of the dominant species will expand by 18.82% and 15.45% under SSP2-4.5 and SSP5-8.5, respectively, with expansion mainly in the northern and western parts of the QTP, shifting overall to higher altitudes (Figure 3b,f, Table 1). In particular, the overlap areas between *E. nutans* and *P. annua*, and between *P. annua* and *S. purpurea*, will shrink, while the area of overlap between *E. nutans* and *S. purpurea* will expand significantly, leading to a shift in niche overlap patterns. Desert grasslands remain fragmented and show no significant change under SSP2-4.5 but tend to contract under SSP5-8.5 (−10.45%) (Figure 3c,g, Table 1). Specifically, the overlap areas between *K. compacta* and *O. abrotanoides* will shrink significantly, and the overlap between *K. compacta* and *S. breviflora* will expand continuously, while *O. abrotanoides* and *S. breviflora* nearly disappear, shifting overall northwest. The overlap in the potential ranges of the three dominant species in temperate grassland expands significantly under SSP2-4.5 and SSP5-8.5, with expansion rates increasing with emission intensity (21.21% and 29.15%, respectively) (Figure 3d,h, Table 1). The areas of overlap between *N. splendens* and *S. bungeana* and between *N. splendens* and *S. krylovii* will expand continuously, primarily in the southern and southeastern parts of the QTP, while the area of overlap between *S. bungeana* and *S. krylovii* will contract continuously.

### 2.3. Changes in the Potential Distribution of Dominant Species from the Current to the Future

**Figure 4:**
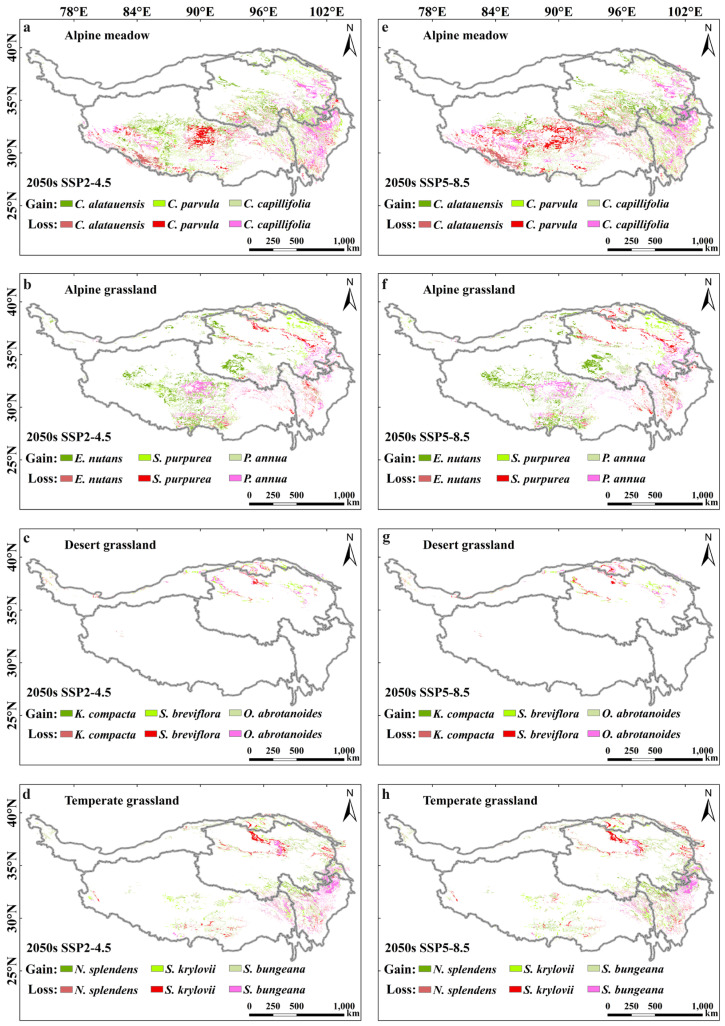
Comparison of changes in the potential distribution of 12 dominant species across different grassland types under future and current climate conditions. The columns in the figure correspond to two SSPs (SSP2-4.5 and SSP5-8.5), with subfigures (**a**–**d**) and (**e**–**h**) representing the four grassland types under each scenario: (**a**,**e**) represent alpine meadow, (**b**,**f**) represent alpine grassland, (**c**,**g**) represent desert grassland, and (**d**,**h**) represent temperate grassland.

Figure 4 illustrates the spatial patterns of distribution expansion and contraction for dominant species of the four grassland types on the QTP under SSP2-4.5 and SSP5-8.5 from the current to the future. The suitable area of *C. alatauensis* will be dominated by expansion, and the expansion areas under SSP2-4.5 and SSP5-8.5 are 10.18 × 10^4^ km^2^ and 9.78 × 10^4^ km^2^, respectively, mainly occurring in the northern and northeastern parts of the QTP (Figure 4a,e, Table 2). However, *C. capillifolia* and *C. parvula* show a contraction trend, which intensifies with higher carbon emission intensity, mainly occurring in the central region of the QTP. In the alpine grassland, the expansion of the suitable habitat of E. nutans is dominant, and this habitat will continue to expand under SSP2-4.5 and SSP5-8.5 in the northwestern QTP (Figure 4b,f, Table 2). The suitable habitats of *P. annua* and *S. purpurea* will continue to shrink, mainly in the southeast of the QTP. For *K. compacta*, *O. abrotanoides* and *S. breviflora* (desert grassland), only a slight expansion is observed along the northern margin under SSP2-4.5, with no obvious contraction; expansion decreases slightly while contraction patches increase marginally under SSP5-8.5 (Figure 4c,g, Table 2). The suitable habitats of *N. splendens*, *S. bungeana*, and *S. krylovii* will continue to expand under SSP2-4.5 and SSP5-8.5. Their expansion trend mainly occurs in the Hengduan Mountains and the southern river valleys, while shrinkage occurs in the northeastern margin of the QTP (Figure 4d,h, Table 2). Overall, under the SSP5-8.5 scenario, the expansion range of the most dominant species is reduced compared to the moderate emission scenario, while contraction trends become more pronounced in some regions, and clear differentiation is observed among the different grassland types.

### 2.4. Centroid Migration of Dominant Species in Different Grasslands on the QTP

**Figure 5:**
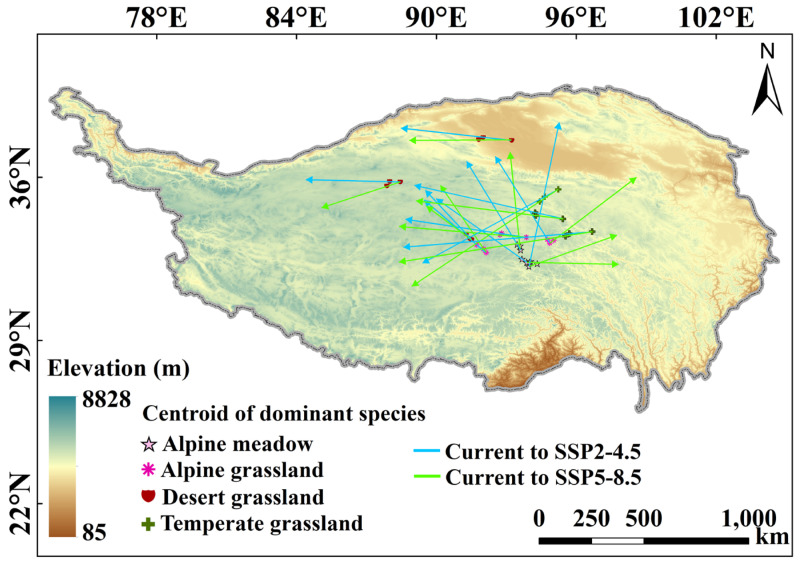
The migration direction of geographical centroids shifting for 12 dominant species across different grassland types on the QTP under SSP2-4.5 and SSP5-8.5. The arrows are only used to indicate the direction of migration.

Our results indicate that the centroid migration directions of dominant species in different grassland types exhibit significant differences under future climate change (Figure 5). The centroids of gravity for dominant species in alpine meadow and alpine grassland will generally migrate northwest under the SSP2-4.5 scenario, whereas this unidirectional trend will diverge under the SSP5-8.5 scenario. Specifically, the centroids of *E. nutans* and *S. purpurea* in alpine grassland will continue to migrate northwest, while alpine meadow species and *P. annua* in alpine grassland will migrate east or northeast. In contrast, the centroids of dominant species in desert grassland will migrate northwest under the SSP2-4.5 and SSP5-8.5 scenarios; among temperate grasslands, the centroids of *N. splendens* and *S. krylovii* will migrate southwest, whereas the centroids of *S. bungeana* will migrate northwest. Overall, the centroid shifts of all dominant species are predominantly toward higher altitudes or higher latitudes on the QTP, which also reflects differences in the sensitivity of dominant species from different grassland types to climate change.

## 3. Discussion

### 3.1. The Reliability of Model Predictions

Plant species are more sensitive to climate change than animals [31]. Understanding how species’ habitats respond to climate change is critical for developing effective conservation strategies [26,32]. We simulated the current and future potential suitable habitats of 12 dominant species from four grassland types on the QTP based on an ensemble model. The model evaluation results showed that all species had AUC > 0.9, TSS > 0.7, and Kappa > 0.6, indicating that the ensemble model predictions have high accuracy and reliability, which is consistent with previous studies [26,27,28,33]. Numerous previous studies have shown that different modeling methods often yield considerably different predictions for the distribution of the same species, whereas ensemble methods, by integrating the outputs of multiple models, can effectively circumvent the biases and uncertainties of single models [23,25,34]. This constitutes the primary rationale for the selection of ensemble modeling in this study.

### 3.2. Analysis of the Contribution of Key Drivers to the Dominant Species on the QTP

This study reveals that the significant spatial differentiation of hydrothermal conditions on the QTP under the current climate leads to a high heterogeneity in the potentially suitable areas for the dominant species of the four grassland types. Meanwhile, the contribution rates of environmental drivers confirmed that elevation plays a dominant role in shaping the geographical patterns of the 12 dominant species, while the combined effects of temperature and precipitation drive the adaptive differentiation of dominant species across different grassland types. This further corroborates the characteristic of elevation-driven hydrothermal heterogeneity in the alpine ecosystems of the QTP [35,36,37]. *C. alatauensis*, *C. capillifolia*, and *C. parvula* are species that prefer humid and cold environments [38,39], but their responses to environmental factors differ markedly among species. The distribution of *C. alatauensis* is mainly regulated by AP, whereas *C. capillifolia* and *C. parvula* are more sensitive to ISO. This may be attributed to their different strategies for alleviating low-temperature stress on the QTP, arising from differences in internal structure and growth microenvironment [40,41]; or it may stem from differences in seed dispersal capacity and limitations in pollinator distribution [42]. The dominant drivers for *E. nutans* and *P. annua* in alpine grassland are AP and PCQ, respectively, while the dominant driver for *S. purpurea* in alpine grassland is MTR. This is because in water-limited alpine grassland, warming may have a negative impact on plant growth, and increased precipitation can alleviate the water stress caused by warming [43], which also explains the regulatory role of precipitation-related factors for most alpine steppe species. The dominant species of desert grassland exhibit a fragmented distribution around the periphery of the Tsaidam Basin, which is closely related to their native environmental characteristics of aridity and water scarcity [44]. The suitable area for the dominant species of temperate grassland shows a pattern of “more in the east, less in the west,” indicating their dependence on warm, humid valleys and lowland environments [45]. The main drivers for *N. splendens* and *S. bungeana* are MTR and MTDQ, respectively, whereas the dominant driver for *S. krylovii* is AP. These differences may arise because temperature and precipitation influence the biogeographic patterns of soil nutrients, thereby leading to variations in the dominant drivers among different species [45,46,47]. These environmentally driven differentiation patterns reveal the mechanism of niche differentiation of dominant grassland species along hydrothermal gradients on the QTP, which confirms the conclusion that the combined effects of elevation, temperature, and precipitation jointly drive niche differentiation.

### 3.3. Impacts of Future Climate Change on the Potential Areas of Dominant Species

Climate change can alter species distribution patterns, and species typically respond to climate change through migration, local adaptation, or extinction [48]. Under the SSP2-4.5 and SSP5-8.5 scenarios, the suitable habitats for the dominant species of four grassland types on the QTP will generally expand to the north, northwest, and high-altitude, high-latitude regions (Figure 4). This finding is consistent with previous studies that show global warming will drive plant expansion toward higher altitudes or higher latitudes [26,49,50,51]. Warmer and wetter conditions favor the expansion of potential habitats for alpine meadow and alpine grassland dominants under SSP2-4.5, while excessive warming under SSP5-8.5 reduces their expansion. In contrast, temperate grassland dominant species show a continuous expansion trend under both scenarios, while suitable areas for desert grassland dominant species remain relatively stable under SSP2-4.5 but exhibit significant contraction under SSP5-8.5. This implies that climate warming will ameliorate cold conditions at high altitudes and high latitudes [52,53], whereas higher temperatures can promote the growth of poisonous weeds [54,55], the hatching and reproduction of pests [10], and exacerbate drought conditions [8], thereby exerting a negative impact on grassland growth.

In addition, we found that the centroid migration of the potential distribution of desert grassland dominant species is consistently northwest under SSP2-4.5 and SSP5-8.5, and the centroids of alpine meadow and alpine grassland dominant species shift northwest under SSP2-4.5, which is consistent with the typical adaptive migration pattern of alpine vegetation in response to warming stress [49,50,51]. However, the centroids of *E. nutans* and *S. purpurea* in alpine grassland will continue to move northwest under SSP5-8.5, while alpine meadow and Poa annua in alpine grassland will migrate east or northeast. This eastward migration may be explained by the fact that rising temperatures will make the eastern and northeastern high-latitude regions cooler and wetter [56,57], thereby providing moisture conditions that compensate for heat stress in cold-wet-preferring species, thus forming new “climate refugia.” Yan and Tang (2019) also show that most endemic species on the QTP are concentrated in the relatively low-altitude zones of the southern and eastern plateau, where conditions are warmer, wetter, and more stable than in the interior plateau [58]. Additionally, the centroid of *S. bungeana* will migrate northwestward. This finding is consistent with the study by Qiao et al. (2020), which reported a northwest shift in the potential distribution of *S. bungeana* under future climate [47]. However, contrary to the general expectation that species migrate to higher altitudes and latitudes in response to climate warming, our results show that the centroids of *N. splendens* and *S. krylovii* will move to the southwest. This phenomenon may be attributed to the complex interaction between rising temperature and precipitation changes. Increased precipitation in the southwestern high-altitude regions of the QTP could partially counteract the negative impacts of elevated temperatures [53,59]. On the other hand, constraints from non-climatic factors, such as soil nutrient limitation, may prevent the formation of stable, suitable habitats even when temperature conditions are favorable [46,60], ultimately driving their migration toward the southwest, where hydrothermal and soil conditions are more suitable. Such interspecific differentiation fundamentally reflects the divergent climate adaptation mechanisms formed by different species based on their own physiological tolerance thresholds, resource utilization strategies, and other factors [61,62]. These findings indicate that species may mitigate the impacts of climate change by shifting their ranges.

In fact, it remains uncertain whether most species will be able to migrate sufficiently rapidly to cope with ongoing climate change [63]. When species fail to keep pace with the velocity of climate change, they lose favorable climatic space and undergo significant range contractions [64,65]. As generation times differ among species, the impacts of climate change rates may vary, and together with variability in genetic variation within populations, these factors may determine their potential for local adaptation [66,67]. Moreover, climate change will affect some species more strongly than others, leading to altered interspecific interactions [68] and potentially exacerbating community instability [69], which may further influence species’ migration capacity. At the same time, alpine plants have a limited ability for seed dispersal and long-distance migration [70], which will directly constrain their migration processes. Furthermore, the projected warming and humidification trend on the QTP will enhance the competitive advantage of these species, thereby affecting community structure and driving shifts in grassland types [10,15,71]. These findings explain the dynamic process of succession of the four grassland types on the QTP from the perspective of species niches; consequently, the predicted changes in grassland types in this study provide theoretical guidance for the rational planning and management of livestock activities. However, this study only analyzed the effects of climate and elevation on the distribution of dominant species of different grasslands, without considering biotic factors such as interspecific relationships and human disturbance or abiotic factors such as soil environment [60,72]; further in-depth research is needed in the future.

## 4. Materials and Methods

### 4.1. Study Area

**Figure 6:**
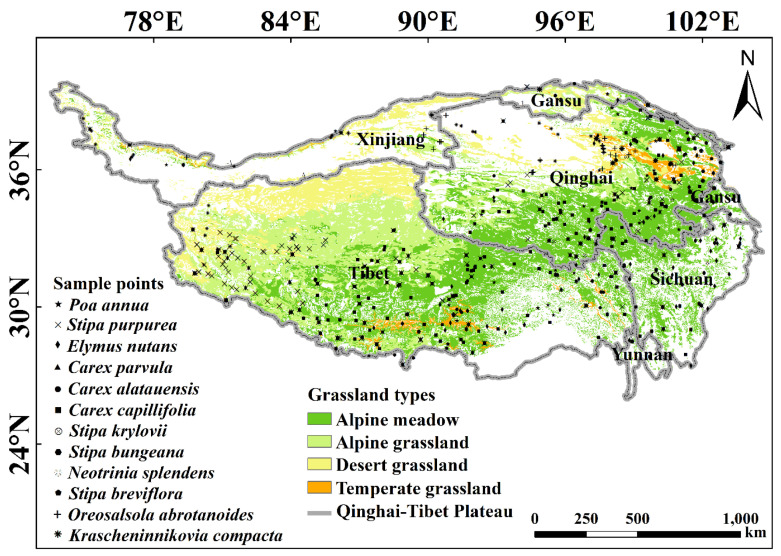
Occurrence data of the 12 dominant species and distribution of grassland types on the QTP.

The QTP (73°19′–104°47′ E, 26°00′–39°47′ N) is located in western China, covering nearly 25% of China’s total area [73]. The annual mean temperature across the QTP ranges from −5.6 to 17.6 °C, with diurnal temperature variations between 14 and 17 °C [74]. From southeast to northwest, the climatic conditions shift from warm and humid to cold and dry [75]. Grasslands are the dominant vegetation type on the QTP, with alpine meadow, alpine grassland, desert grassland, and temperate grassland together accounting for 73.6% of the total area of the QTP [10] (Figure 6). The 12 dominant species used in this study include *Carex alatauensis*, *Carex capillifolia* and *Carex parvula* for the alpine meadow [76]; *Elymus nutans*, *Poa annua* and *Stipa purpurea* for alpine grassland [10,77]; *Krascheninnikovia ceratoides*, *Oreosalsola abrotanoides* and *Stipa breviflora* for desert grassland [44,78]; and *Neotrinia splendens*, *Stipa bungeana* and *Stipa krylovii* for temperate grassland [45,46,79].

### 4.2. Data Sources

#### 4.2.1. Species Distribution Data

Species occurrence data were primarily obtained from the field survey conducted by Jin et al. (2022) on the QTP during 2018–2021 [80]; the National Specimen Information Infrastructure (NSII, http://www.nsii.org.cn); the Chinese Field Herbarium (CFH, http://www.cfh.ac.cn); and the Global Biodiversity Information Facility (GBIF, https://www.gbif.org) (GBIF.org, GBIF Occurrence Download. Available online: https://doi.org/10.15468/dl.jkt9af (accessed on 2 may 2026)). Specimen information was verified online through the Chinese Virtual Herbarium (CVH, https://www.cvh.ac.cn). The longitude, latitude, and species name of each species occurrence record were saved in CSV format. In addition, to reduce spatial autocorrelation and sampling bias and to ensure a more even distribution of occurrence points, we removed duplicate records and applied spatial filtering, retaining only one occurrence record within a 1 km radius. Specifically, we defined a 1 km radius around each sampling point and removed all other points within that circle; points other than the center of the circle were deleted. Finally, we obtained 1214 valid occurrence records for 12 species. Among them, *C. alatauensis* (99), *C. capillifolia* (213), *C. parvula* (215), *E. nutans* (178), *P. annua* (50), *S. purpurea* (176), *K. ceratoides* (45), *O. abrotanoides* (38), *S. breviflora* (55), *N. splendens* (49), *S. bungeana* (54), and *S. krylovii* (42) (Figure 6).

#### 4.2.2. Environmental Data and Preprocessing

Elevation data and current (representative 1970–2000) and future (2050: average of 2041–2060) bioclimatic variables (Table S1 provides the specific names and their meanings) were downloaded from WorldClim 2.1 (http://www.worldclim.org/) with a spatial resolution of 30″ [81]. To account for future climate uncertainty, we used 2050 climate data based on the BCC-CSM2-MR Global Climate Model (GCM), which has been widely applied in species distribution modeling on the QTP [82,83]. In addition, we consider two Shared Socioeconomic Pathways (SSPs) scenarios [84,85,86]: SSP2-4.5 and SSP5-8.5, where the number after SSP represents the conversion of the warming effect of carbon dioxide into an equivalent wattage of radiation gain. SSP2-4.5 represents a “middle way” model of social, economic and technological trends throughout the twenty-first century that does not deviate significantly from historical development (with a radiative forcing of about 4.5 W/m^2^); SSP5-8.5, which represents rapid global economic and social development alongside a shift toward resource- and energy-intensive lifestyles, is estimated to result in a radiative forcing of about 8.5 W/m^2^ in the year 2100.

Spatial autocorrelation among environmental variables may affect the analysis of the relationship between species distribution and environment [87]. To avoid multicollinearity among the environmental variables, we conducted a correlation analysis using the Spearman correlation coefficient to examine the relationship between 19 climate factors and elevation at the species locations (Figure S1). If the absolute correlation coefficient |r| ≥ 0.7, the two variables were considered highly correlated. Therefore, we removed variables with a correlation higher than 0.7 [88], and the final ensemble model considered seven variables (Table 3).

### 4.3. Ensemble Model Construction and Performance Evaluation

Ensemble methods, which combine multiple single models, can effectively reduce the uncertainty associated with different model predictions and are widely used in species distribution modeling [24,89]. To simulate the potential suitable habitats of dominant species in four grassland types under the future climate, we constructed an ensemble model using a weighted average approach, incorporating three ENMs: maxent, random forest (RF), and support vector machine (SVM). Among them, Maxent predicts occurrence probability by modeling the nonlinear relationship between species occurrence points and environmental factors [90]. In this paper, it was run using the default parameters of the “dismo” package in R (version 4.4.1). RF constructs an ensemble decision tree by bootstrap resampling (we set ntree = 1000 and implement it using the “randomForest” function) and avoids overfitting by randomly selecting samples and splitting attributes [91,92]. SVM projects the data into a high-dimensional feature space for pattern classification [93]. We use a radial basis kernel function (based on the “e1071” package in R) and perform parameter tuning based on 10-fold cross-validation. The weight ($w_{i}$) of each model was calculated based on the area under the receiver operating characteristic curve (AUC) value according to the formula $w_{i}=AUC_{i}/\sum_{i=1}^{3}AUC_{i}$. Subsequently, we used the “randomPoints” function to randomly generate pseudo-absence data in the study area at a ratio of presence to pseudo-absence of 1:1 [94]. Among them, the ensemble model was built using 80% of the randomly selected data, with the remaining 20% used to evaluate the current predictive performance of the model. As the AUC is often considered insufficient for evaluating the performance of species distribution models [95], we evaluated the accuracy of model predictions using three metrics: AUC, True Skill Statistic (TSS), and Cohen’s Kappa (Kappa) [96,97]. The higher values of AUC, TSS, and Kappa indicate stronger model performance and greater reliability of the results. Generally, AUC values between 0.7 and 0.8 are considered general, 0.8–0.9 are considered good, and 0.9–1.0 are considered excellent; TSS values between 0.6 and 0.8 are considered good, and 0.8–1.0 are considered excellent; Kappa values between 0.4 and 0.75 are considered good, and values greater than 0.75 are considered excellent [81,98].

### 4.4. Changes in the Potential Distribution of Dominant Species and Centroid Migration

In this study, we used the sum of maximizing sensitivity and specificity to determine the threshold [99], which is unaffected by the prevalence of the data used to build the model and tends to favor sensitivity (true presence) over specificity (true absence) [100]. Subsequently, we divided the suitable habitats (low, medium, and high) and unsuitable habitats for species distribution based on this threshold. Furthermore, to facilitate the comparison of suitable habitat changes for different species between current and future (SSP2-4.5 and SSP5-8.5), we converted the continuous probabilities into binary data (0–1) according to the threshold, where values below the threshold were assigned 0 (i.e., absence) and values above the threshold were assigned 1 (i.e., presence), thereby visualizing the changes in species spatial distribution patterns under future climate change. Calculated according to [current + 2 (future + 1)] − 2, the result has four scenarios: 0 represents the unsuitable habitats; 1 indicates the distribution that will be lost under future climate change; 2 indicates the distribution that will be gained under future climate change; 3 indicates stable distributions under future climate change. Moreover, we obtained the potential spatial overlap area of the three dominant species in the same grassland type by superimposing the independent distribution map layers of the three dominant species in the same grassland type.

In addition, shifts in the geographic center of species distribution over time can reflect the overall trajectory of its range change [101,102]. We assumed the study area constitutes a homogeneous plane, where the point at which the species distribution reaches a balance in terms of torque on this plane is the geographic center of the species distribution [103]. Let $P_{ij}$ be the probability of survival in terms of environmental adaptation for the patch (*i*, *j*) predicted by the ENM, and $N_{i}$ and $E_{j}$ represent the latitude and longitude of the center of patch (*i*, *j*), respectively. We calculated the geographic distribution center’s latitude (*N*) and longitude (*E*) by setting $N=(\sum_{j=1}^{n}P_{ij}\times N_{i})/\sum_{i=1}^{n}\sum_{j=1}^{m}P_{ij}$ and $E=(\sum_{i=1}^{n}P_{ij}\times E_{j})/\sum_{i=1}^{n}\sum_{j=1}^{m}P_{ij}$. Meanwhile, we calculated the geographic distance between two centers using the following formula:

$$D=r\times 2\arcsin(\sqrt{\sin^{2}(\frac{a}{2})+\cos(x_{1})\times \cos(x_{2})\times \sin^{2}(\frac{b}{2})})$$

where $\left(x_{1},y_{1}\right)$ and $\left(x_{2},y_{2}\right)$ represent the latitudes and longitudes of centers A and B, respectively, and *r* is the Earth radius, which takes the value of 6378.137 km, with $a=x_{1}-x_{2}$ and $b=y_{1}-y_{2}$.

## 5. Conclusions

This study used an ensemble model to simulate the potential distribution of 12 dominant species across four grassland types (alpine meadow, alpine grassland, desert grassland, and temperate grassland) on the QTP under current and future climate scenarios and systematically revealed the spatial pattern dynamics of the suitable habitats of these dominant species and their driving mechanisms. The main findings are as follows: (1) The ensemble model showed excellent predictive accuracy for the 12 dominant species (AUC > 0.9, TSS > 0.7, Kappa > 0.6). (2) Elevation is the key environmental factor affecting their distribution, and the combined effects of temperature and precipitation dominate the niche differentiation among different grassland types, reflecting the adaptive characteristics of dominant species in different grassland types along the hydrothermal gradient. (3) The current and future potential distributions of the dominant species exhibit clear spatial differentiation along the southeast–northwest hydrothermal gradient of the QTP, and the contraction of suitable habitats for all species will intensify with increasing carbon emissions. (4) The direction of centroid migration varies among dominant species of different grassland types. Specifically, the centers of gravity of the desert grassland and *S. bungeana* will migrate to the northwest, while *N. splendens* and *S. krylovii* will migrate to the southwest; the alpine meadow and alpine grassland will migrate to the northwest under the SSP2-4.5 scenario and diverge under the SSP5-8.5 scenario. Overall, this study overcomes the limitations of previous studies that focused on a single species or a single grassland type; deduces the overall evolution of grassland types from the distribution changes of dominant species; quantifies the spatial response patterns of QTP grasslands under climate change; and provides scientific theoretical support for the conservation of alpine ecosystems, the maintenance of biodiversity, and adaptive grassland management.

## Figures and Tables

**Table 1:** The potential distribution areas of dominant species of different grassland types under current and future climate scenarios (I, II, and III represent the dominant species in different grassland types, where I represents *C. alatauensis* (alpine meadow), *E. nutans* (alpine grassland), *K. compacta* (desert grassland), and *N. splendens* (temperate grassland); II represents *C. capillifolia* (alpine meadow), *P. annua* (alpine grassland), *O. abrotanoides* (desert grassland) and *S. bungeana* (temperate grassland); III represents *C. parvula* (alpine meadow), *S. purpurea* (alpine grassland), *S. breviflora* (desert grassland) and *S. krylovii* (temperate grassland)).

Type of Grassland	Climate Scenarios	Overlap Area of Potential Distribution (×10^4^ km^2^)			
		I and II	I and III	II and III	I, II and III
Alpine meadow	current	1.93	36.72	9.29	142.1
	SSP2-4.5	2.76	39.86	3.3	163.88
	SSP5-8.5	2.45	40.85	2.57	155.73
Alpine grassland	current	64.89	22.71	6.81	105.57
	SSP2-4.5	57.48	29.89	4.14	125.44
	SSP5-8.5	51.99	32.21	3.38	121.88
Desert grassland	current	21.74	13.9	0.012	17.32
	SSP2-4.5	13.87	18.99	0.0002	17.36
	SSP5-8.5	14.55	19.96	0.00	15.51
Temperate grassland	current	20.44	14.18	7.93	74.44
	SSP2-4.5	30.65	27.74	6.29	90.23
	SSP5-8.5	26.33	30.79	4.92	96.14

**Table 2:** Area changes in the potential distribution of dominant species in different grassland types from the current to the future (I, II, and III represent the dominant species in different grassland types, where I represents *C. alatauensis* (alpine meadow), *E. nutans* (alpine grassland), *K. compacta* (desert grassland), and *N. splendens* (temperate grassland); II represents *C. capillifolia* (alpine meadow), *P. annua* (alpine grassland), *O. abrotanoides* (desert grassland) and *S. bungeana* (temperate grassland); III represents *C. parvula* (alpine meadow), *S. purpurea* (alpine grassland), *S. breviflora* (desert grassland) and *S. krylovii* (temperate grassland)).

Type of Grassland	Climate Scenarios	Changes the Current to the Future (×10^4^ km^2^)					
		I Gain	I Loss	II Gain	II Loss	III Gain	III Loss
Alpine meadow	SSP2-4.5	10.18	2.52	11.41	12.5	5.82	8.06
	SSP5-8.5	9.78	3.02	10.5	13.98	5.07	9.89
Alpine grassland	SSP2-4.5	12.77	0.06	0.48	7.77	3.85	4.21
	SSP5-8.5	12.84	0.03	0.28	8.7	2.73	4.44
Desert grassland	SSP2-4.5	0.01	0.2	0.13	1.88	1.87	0.9
	SSP5-8.5	0.003	0.11	0.21	1.31	1.42	1.21
Temperate grassland	SSP2-4.5	2.79	2.28	7.59	7.18	3.2	2.95
	SSP5-8.5	4.51	2.29	6.61	6.12	3.54	3.31

**Table 3:** Environment variables for modeling.

Symbol	Meaning of Variables
MTR	Mean monthly temperature range/°C
ISO	Isothermality
MTDQ	Mean temperature of the driest quarter/°C
AP	Annual precipitation/mm
PS	Precipitation seasonality
PCQ	Precipitation of the coldest quarter/mm
Elev	Elevation

## Data Availability

The original contributions presented in this study are included in the article/Supplementary Materials. Further inquiries can be directed to the corresponding author.

## Supplementary Materials

The following supporting information can be downloaded at https://www.mdpi.com/article/10.3390/plants15131972/s1, Figure S1. Spearman correlation analysis of environment variables. Table S1. Meaning of 19 bioclimatic factors and topographical variables.
