# Supplementary material for: Centroid Migration and Distribution of Dominant Species in Different Grassland Types Revealing Climate Change Responses on the Qinghai–Tibet Plateau
Source: Plants (Basel). 2026 Jun 26;15(13):1972. doi: 10.3390/plants15131972 (PMC13363827; doi:10.3390/plants15131972)
Supplement: Supplementary file 1 [file plants-15-01972-s001.zip › plants-4380145-supplementary.pdf]

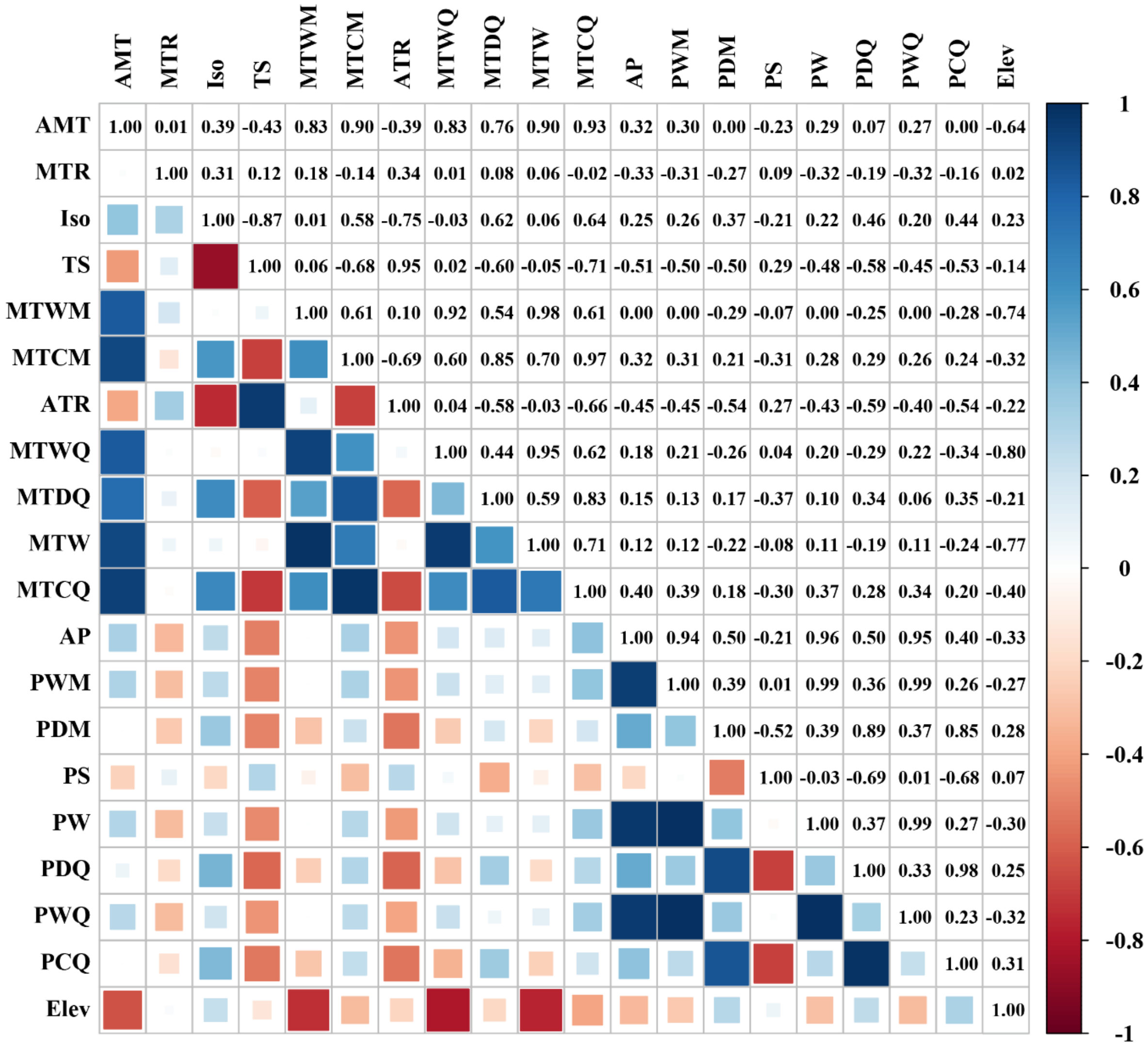

Figure S1. Spearman correlation analysis of environment variables.

Table S1. Meaning of 19 bioclimatic factors and topographical variables.

Symbol	Climatic variables
AMT	Annual mean temperature/°C
MTR	Mean monthly temperature range/°C
Iso	Isothermality
TS	Temperature seasonality
MTWM	Max temperature of warmest month/°C
MTCM	Min temperature of coldest month/°C
ATR	Annual temperature range/°C
MTWQ	Mean temperature of wettest quarter/°C
MTDQ	Mean temperature of driest quarter/°C
MTW	Mean temperature of warmest quarter/°C
MTCQ	Mean temperature of coldest quarter/°C
AP	Annual precipitation/mm
PWM	Precipitation of wettest month/mm
PDM	Precipitation of driest month/mm
PS	Precipitation seasonality
PW	Precipitation of wettest quarter/mm
PDQ	Precipitation of driest quarter/mm
PWQ	Precipitation of warmest quarter/mm
PCQ	Precipitation of coldest quarter/mm
Elev	Elevation/m
